# Supplementary figures and images for: Brucella proline racemase protein A targets Tpl2 to promote IL-10 secretion for establishment of chronic infection
Source: Front Immunol. 2026 Jun 9;17:1808256. doi: 10.3389/fimmu.2026.1808256 (PMC13320626; doi:10.3389/fimmu.2026.1808256)

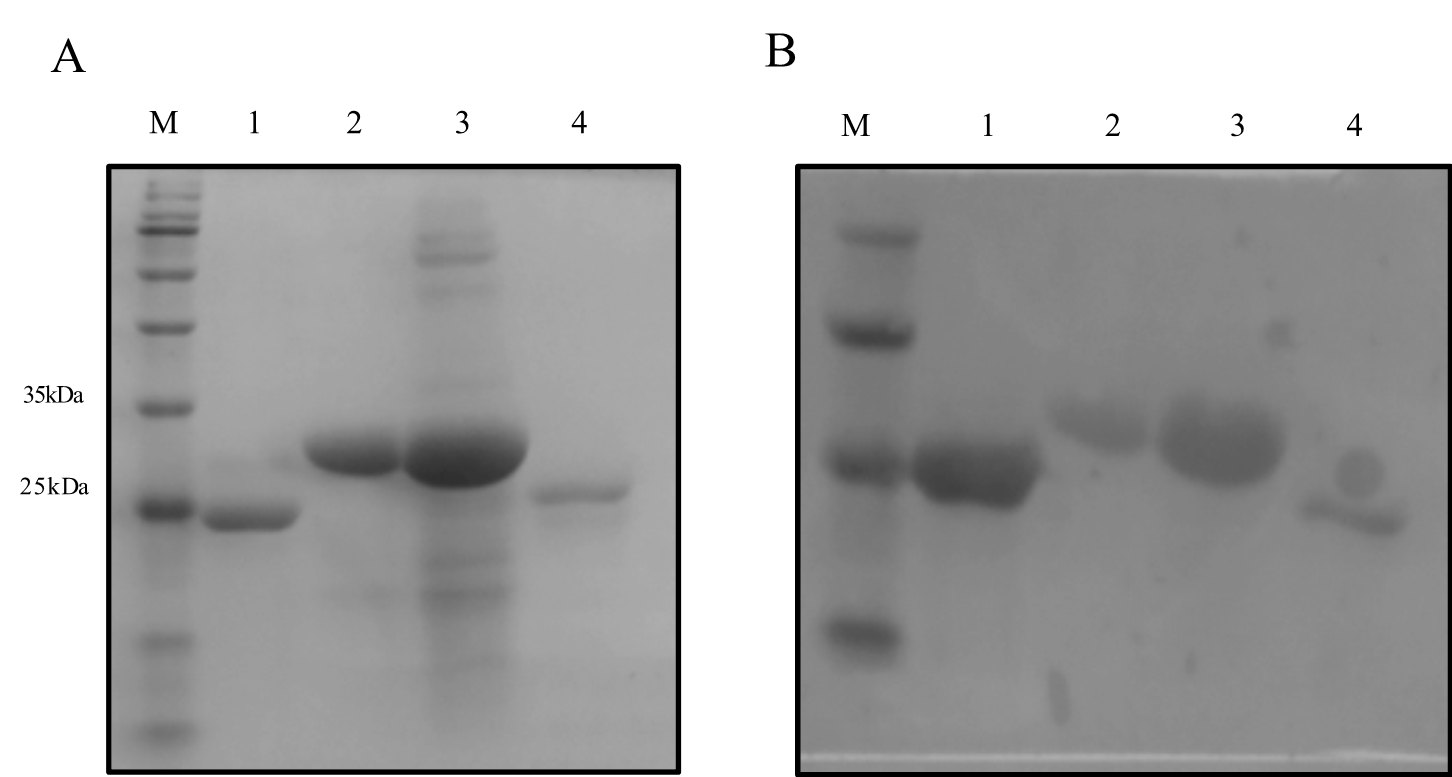

Supplement: Supplementary Figure 1 — Expression and Purification of rOMP25, rRomA, rPrpA, and rwadC (A) Analysis of the four recombinant proteins using SDS-PAGE. Fifteen micrograms of each protein was loaded onto SDS-PAGE. (B) Analysis of the four recombinant proteins using western blotting with anti-His antibody. Lane M: molecular weight marker (CWBIO, Beijing, China), lane 1, outer membrane protein 25; lane 2, RomA protein; lane 3, PrpA protein; lane 4, wadC protein. [file Image1.tiff]

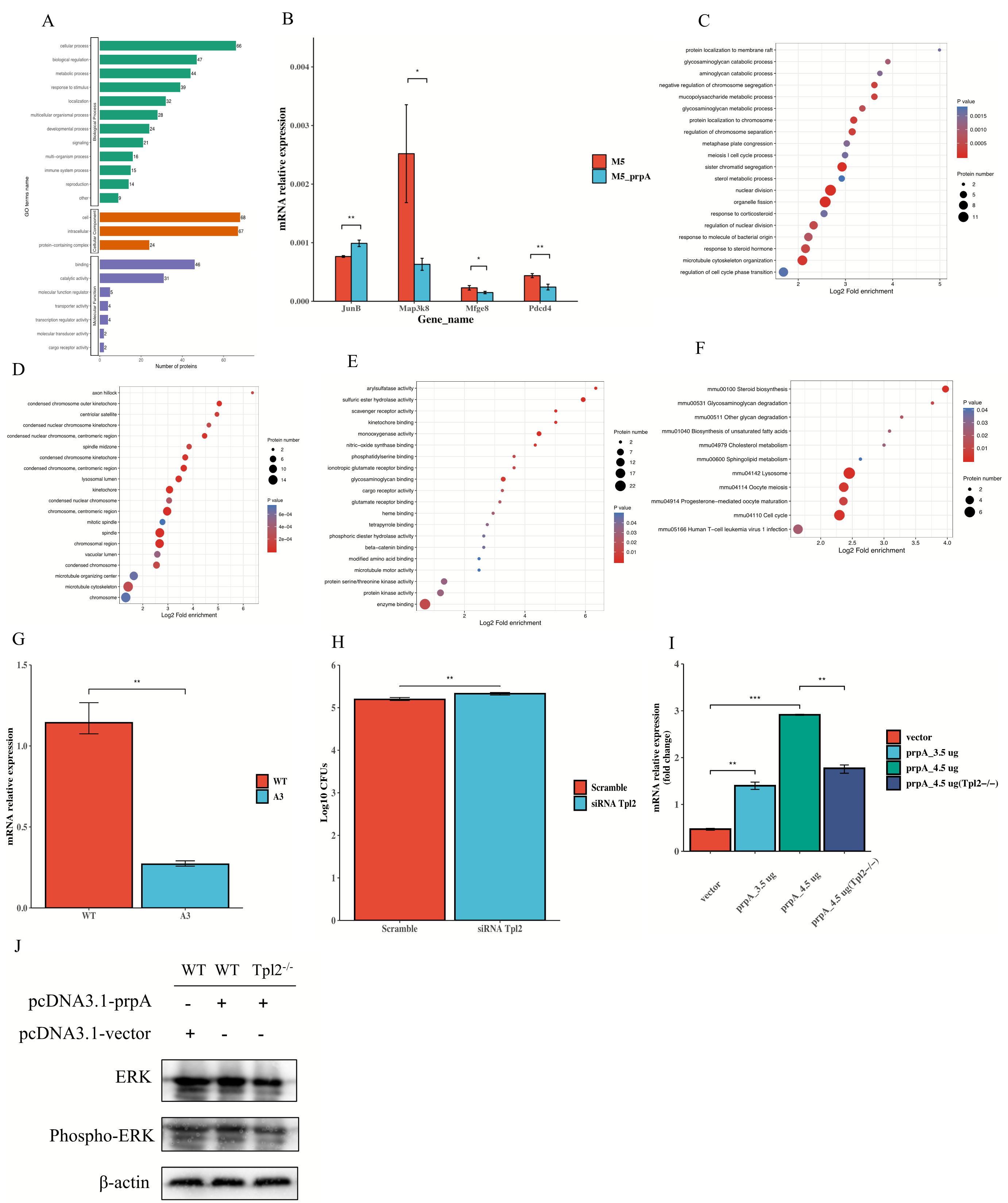

Supplement: Supplementary Figure 2 — Analysis of Tpl2 Function in the MAPK Signaling Pathway During Brucella Infection (A) Analysis of differentially expressed proteins through the GO database. (B) Identification of key genes associated with IL-10 production in macrophages. (C) Biological processes of DE proteins. (D) Cellular component of DE proteins. (E) Molecular functions of DE proteins. (F) KEGG pathway enrichment results of DE proteins. (G) Identification of Tpl2 expression levels in wild-type or Tpl2-/- macrophages using RT-qPCR. (H) Enumeration of CFUs in wild-type or macrophages expressing Tpl2 after interference with siRNA, followed by infection with B. melitensis M5–90 at 24 h post-infection (PI). (I) Identification of ERK gene expression levels in wild-type or Tpl2-/- macrophages using RT-qPCR. RAW264.7 cells or Tpl2-/- RAW264.7 cells were transfected with pcDNA3.1-prpA or vector, total RNA was isolated, and cDNA was synthesized. ERK expression was analyzed using RT-qPCR, and gene expression levels were normalized to GAPDH. (J) Identification of ERK expression in wild-type or Tpl2-/- macrophages using western blotting. Wild-type or Tpl2-/- macrophages were transfected with indicated plasmids, and ERK or phosphor-ERK expression was identified using western blotting with anti-ERK1/2 or anti-phospho-ERK1/2 antibody. [file Image2.tiff]

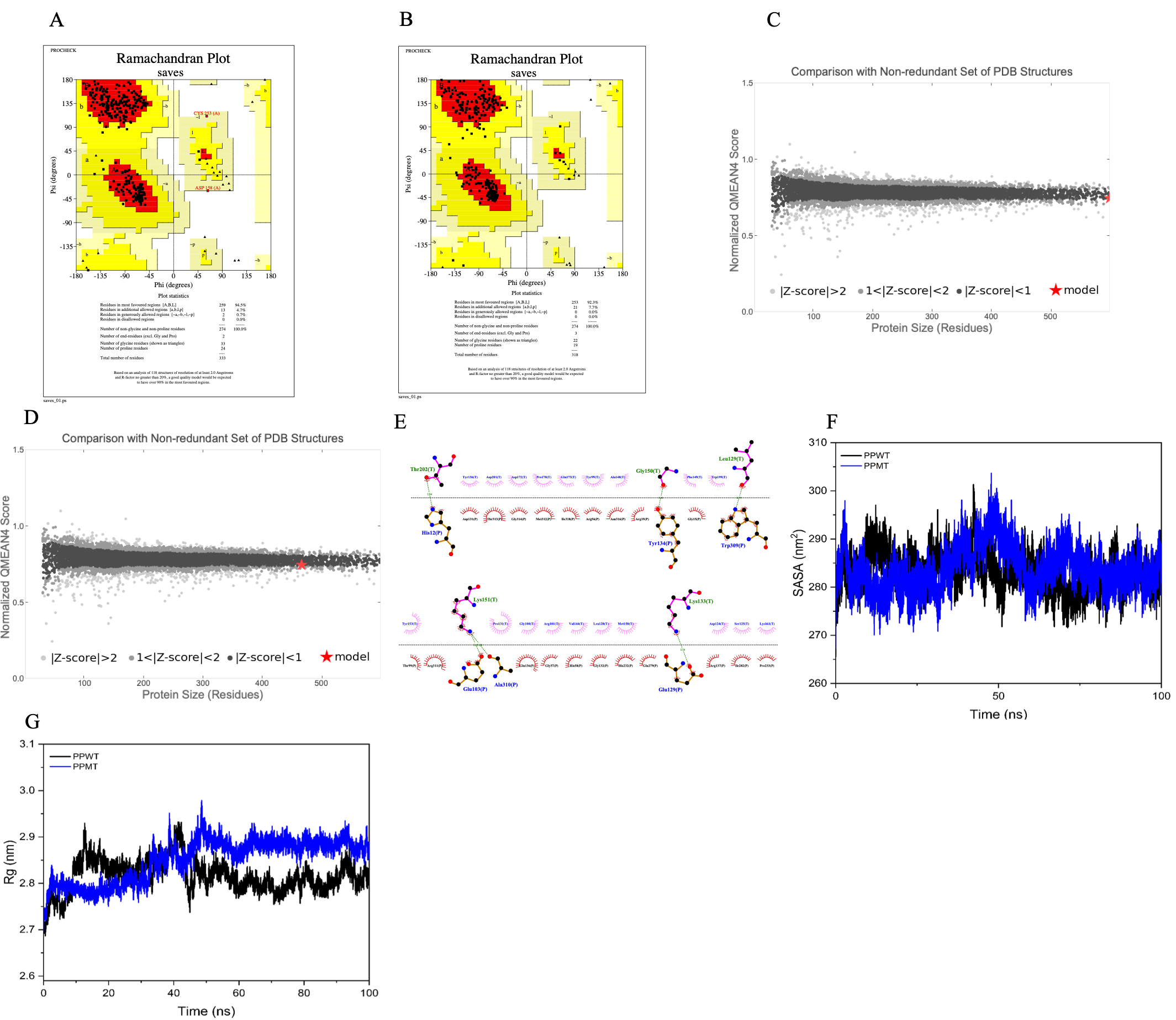

Supplement: Supplementary Figure 3 — Examination of the PrpA and Tpl2 Model (A, B) Ramachandran plot analysis of the PrpA (A) and Tpl2 (B) models. (C, D) Z-scores of all structures in the PDB database, where the PrpA (PDB ID: 1w61) and Tpl2 (PDB ID: 5iu2) models marked in red pentastar have Z-scores < 1. (E) Two-dimensional docking results between PrpA and Tpl2. In the figure, T represents Tpl2, and P represents PrpA. (F, G) Analysis of solvent-accessible surface area (SASA) and radius of gyration (Rg) in molecular dynamics simulations of the PrpA-Tpl2 system or PrpA-mutant-Tpl2 system. [file Image3.tiff]

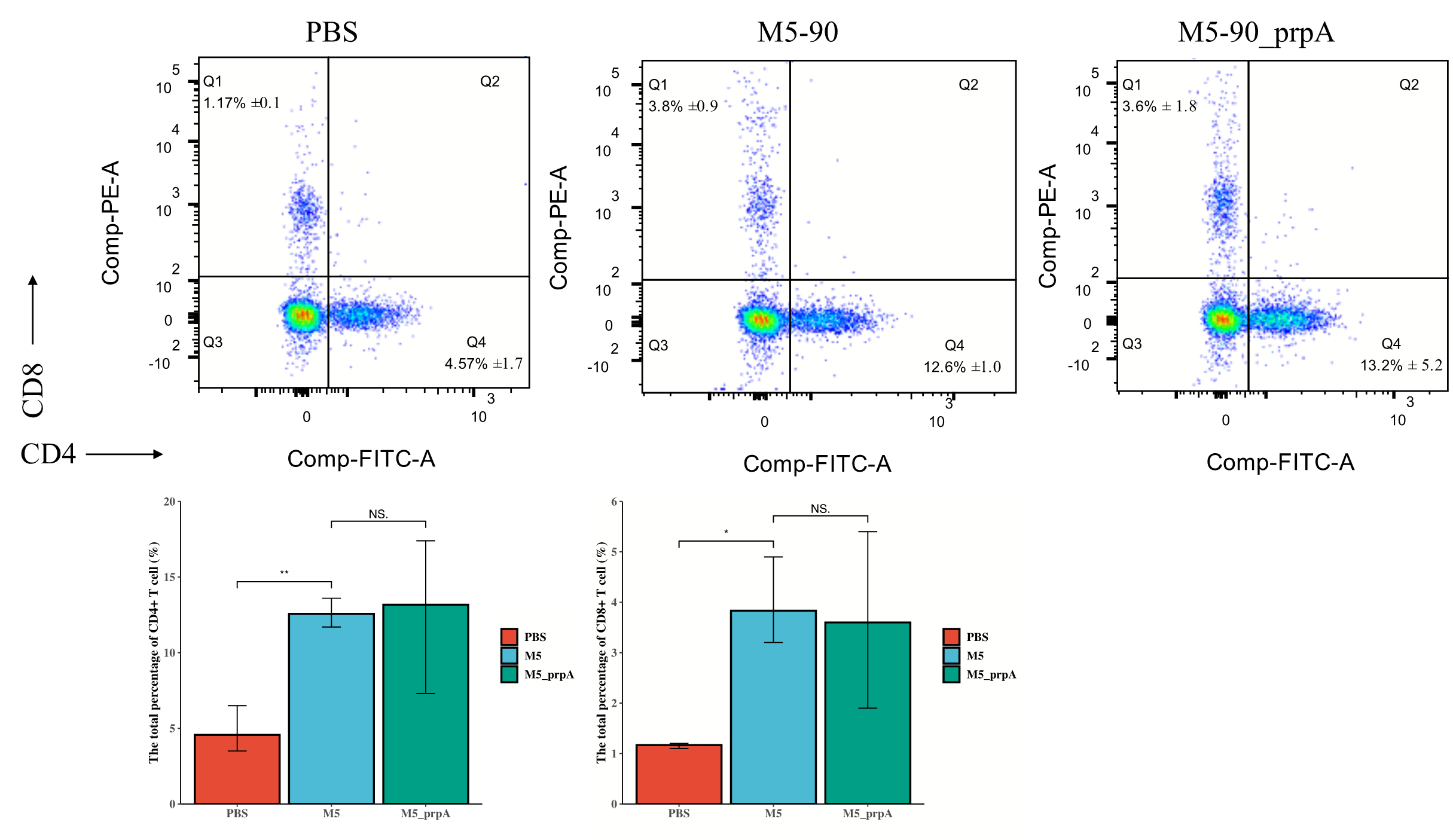

Supplement: Supplementary Figure 4 — Determination of the number of the CD4+ and CD8+ T cells in mice immunized with B. melitensis M5-90, B. melitensis M5–90 prpA mutant or PBS (Negative control) for 45 days. [file Image4.tiff]

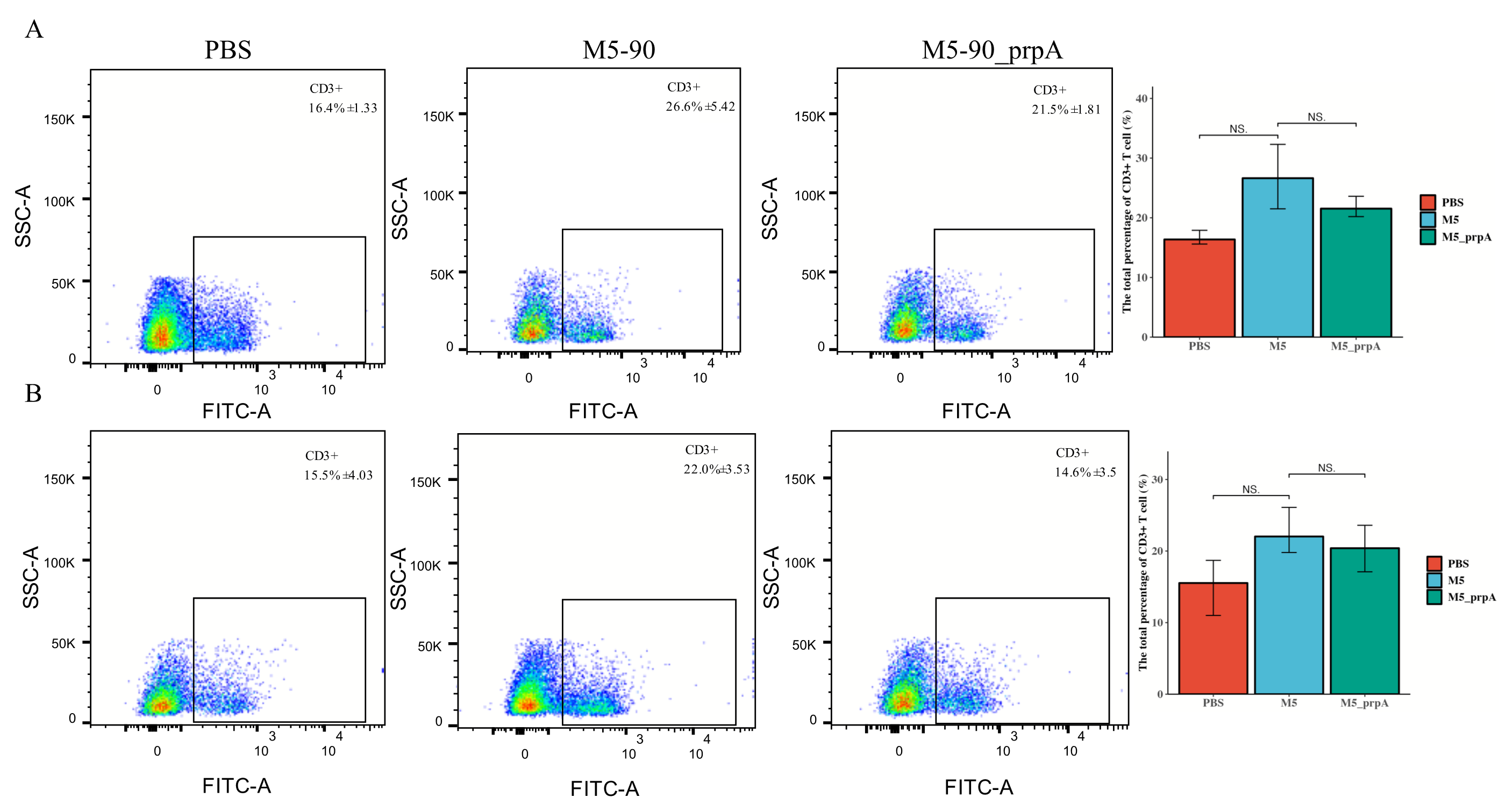

Supplement: Supplementary Figure 5 — Determination of the number of the CD3+ T cells in mice immunized with B. melitensis M5-90, B. melitensis M5–90 prpA mutant or PBS (Negative control) for 30 (A) and 45 days (B). [file Image5.tiff]
