# Supplementary material for: Brucella proline racemase protein A targets Tpl2 to promote IL-10 secretion for establishment of chronic infection
Source: Front Immunol. 2026 Jun 9;17:1808256. doi: 10.3389/fimmu.2026.1808256 (PMC13320626; doi:10.3389/fimmu.2026.1808256)
Supplement: Supplementary file 6 [file Table1.docx]

**Supplementary Table 1.** Primers for construction of *B. melitensis* M5-90 *prpA* mutant

| Gene name | Primer sequence (5'– 3') | Size (bp) |
| --- | --- | --- |
| prpA-N-F | TTCATTTTATTCCCGGTCCATTT | 234 |
| prpA-N-R | AGTGGGTATAACAGGCGAAATTA |  |
| prpA-C-F | TATGGTCAGTTCCGAAAACGTTG | 237 |
| prpA-C-R | CCTTAAGGTTGAAGGCGGCAGGC |  |
| SacB-F | GGGCTGGAAGAAGCAGACCGCTA | 1423 |
| SacB-R | GCTTATTGTTAACTGTTAATTGTCC |  |
| B. melitensis M5-90 *prpA* mutant_identification-F | TTATGCCACGCTGAACCCATGAGC | 1002 |
| B. melitensis M5-90 *prpA* mutant_identification-R | ATGGCAAGACATTCCTTCTTCTG |  |

**Supplementary Table 2.** Primers for construction of the recombinant plasmids.

| Gene name | Primer sequence (5'– 3') | Size (bp) |
| --- | --- | --- |
| pET32a-prpA-F | GAATTCTTATGCCACGCTGAACCCATGAGC | 1002 |
| pET32a-prpA-R | CTCGAGATGGCAAGACATTCCTTCTTCTGCG |  |
| pET28a-omp25-F | GAATTCATGCGCACTCTTAAGTCTCTCGTA | 642 |
| pET28a-omp25-R | CTCGAGTTAGAACTTGTAGCCGATGCCGAC |  |
| pET32a-wadC-F | GAATTCCCAACTTCAAGCGCCGCCTTTC | 879 |
| pET32a-wadC-R | CTCGAGCGATGGCGTTCGTCAATGCGCG |  |
| pET32a-RomA-F | GAATTCATCAGAGATCGACGTCGAGAAT | 256 |
| pET32a-RomA-R | CTCGAGATGCCGGGTGCAATAAACCGGC |  |
| pGEX-4T-1-Tpl2-F | GAATTCATGGAATACATGAGCACCGGTAG | 1416 |
| pGEX-4T-1-Tpl2-R | CTCGAGTTAACCATATTCCAGGGTCGG |  |

The underlined nucleotides represents the restriction enzyme sites.

**Supplementary Table 3.** Primers for construction of eukaryotic expression plasmids.

| Gene name | Primer sequence (5'– 3') | Size (bp) |
| --- | --- | --- |
| prpA-F | GAATTCTTATGCCACGCTGAACCCATGAGC | 1002 |
| prpA-R | CTCGAGATGGCAAGACATTCCTTCTTCTGCG |  |
| Tpl2-F | GAATTCAGTGCAGGCCTGGGCTGTGGCGC | 2507 |
| Tpl2-R | CTCGAGAAGACAAAATACTCAGTTTATTT |  |

The underlined nucleotides represents the restriction enzyme sites.

**Supplementary Table 4.** Primers used for RT-qPCR in this study.

| Gene name | Primer sequence (5'– 3') | Size (bp) |
| --- | --- | --- |
| c-fos-F | CGGGTTTCAACGCCGACTA | 166 |
| c-fos-R | TTGGCACTAGAGACGGACAGA |  |
| c-maf-F | CTTTGAGGCCATCAACTCACA | 243 |
| c-maf-R | CTGCACTTGTCGCTCAGAG |  |
| p38-F | GGGACACCCCCTGCTTATCT | 275 |
| p38-R | TCCCTGCTTTCAAAGGACTGG |  |
| NF-κBp65-F | AGGCTTCTGGGCCTTATGTG | 111 |
| NF-κBp65-R | TGCTTCTCTCGCCAGGAATAC |  |
| STAT3-F | CAATACCATTGACCTGCCGAT | 109 |
| STAT3-R | GAGCGACTCAAACTGCCCT |  |
| Tpl2-F | ATGGAGTACATGAGCACTGGA | 116 |
| Tpl2-R | GGCTCTTCACTTGCATAAAGGTT |  |
| ERK-F | GGTTGTTCCCAAATGCTGACT | 84 |
| ERK-R | CAACTTCAATCCTCTTGTGAGGG |  |
| JunB-F | CGACGGTTTTGTCAAAGCCC | 118 |
| JunB-R | CTTGCTTGAGCTGCGCCACT |  |
| Mfge8-F | TACATGTT CACCACAGCCGT | 109 |
| Mfge8-R | TGCTGTGTAGAAC AACGGGAG |  |
| Pdcd4-F | TGACATCCAAGCTGCTTTCTG | 104 |
| Pdcd4-R | AGATCCTTCAGCAACTTGTCA |  |
| GAPDH-F | AGGTCGGTGTGAACGGATTTG | 123 |
| GAPDH-R | TGTAGACCATGTAGTTGAGGTCA |  |

**Supplementary Table 5.** The sequences of siRNA used in this study.

| Gene name | Primer sequence (5'– 3') |
| --- | --- |
| *c-fos* siRNA | CGAAAGGGCAGCAGCAGCATT |
|  | UGCUGCUGCUGCCCUUUCGTT |
| *c-maf* siRNA | ACGAGAAGCUGGUGAGCAATT |
|  | UUGCUCACCAGCUUCUCGUTT |
| *p38* siRNA | GGUCACUGGAGGAAUUCAAUG |
|  | UUGAAUUCCUCCAGUGACCUU |
| *NF-κBp65* siRNA | CAGUAUUCCUGGCGAGAGA |
|  | UCUCUCGCCAGGAAUACUG |
| *STAT3* siRNA | GGAGAAGCAUUGUGAGUGATT |
|  | UCACUCACAAUGCUUCUCCTT |
| *Tpl2* siRNA | GGCUGUCAUCUGUCAGAUAUG |
|  | UAUCUGACAGAUGACAGCCAG |

**Supplementary Table 6.** The binding energy of PPWT and PPMT predicted using MMGBSA.

| Type | PPWT (kJ/mol) | PPMT(kJ/mol) |
| --- | --- | --- |
| *E_VDW_* | -101.48 +/- 6.86 | -82.95 +/- 5.61 |
| *E_ELE_* | -55.78 +/- 8.15 | -75.3 +/- 5.36 |
| *E_GB_* | 55.98 +/- 2.16 | 75.33 +/- 3.16 |
| *E_SA_* | -13.88 +/- 0.83 | -11.46 +/- 0.74 |
| *G_binding energy_* | -115.16 +/- 8.87 | -94.37 +/- 8.41 |

*E_VDW_*: van der Waals energy

*E_ELE_*: eletrostatic energy

*E_GB_*: eletrostatic contribution to solvation

*E_SA_*: non-polar contribution to solvation
